# The effectiveness of home-based therapy on functional outcome, self-efficacy and anxiety among discharged stroke survivors

**DOI:** 10.1097/MD.0000000000023296

**Published:** 2020-11-20

**Authors:** Chong Pui Kei, Nor Azlin Mohd Nordin, Aznida Firzah Abdul Aziz

**Affiliations:** a Physiotherapy Program, Center for Rehabilitation and Special Needs Studies, Faculty of Health Sciences, Universiti Kebangsaan Malaysia; b Physiotherapy Unit, Hospital Rehabilitasi Cheras; c Department of Family Medicine, Faculty of Medicine, Universiti Kebangsaan Malaysia, Cheras Kuala Lumpur, Malaysia.

**Keywords:** anxiety, gait speed, self-efficacy, stroke

## Abstract

**Introduction:**

Stroke survivors are commonly at risk of functional decline following discharge from rehabilitation, which increase their susceptibility to falls, dependency in activities of daily living and emotional disturbances. To combat these, continued therapy is important. Home-based therapy (HBT) has been shown to be useful in maintaining functional performance and quality of life of chronic stroke survivors. However, evidence on its effectiveness remains limited, while no studies are available to date which report the benefit of HBT on stroke survivors self-efficacy and emotional status. Therefore, this study aims to assess the effectiveness of post-discharge HBT in comparison to usual practice on functional outcome (mobility and gait speed), self-efficacy and anxiety level among stroke survivors.

**Methods:**

This is an assessor-blinded randomized control trial comparing 2 types of intervention which are HBT (experimental group) and usual practice (UP) (control group). Based on sample size calculation using GPower, a total number of 42 participants will be recruited and allocated into either the HBT or the UP group. Participants in HBT group will receive a set of structured exercise therapy consisting of progressive strengthening, balance and task-related exercises. While participants in UP group will receive a usual “intervention” practised by rehabilitation professional prior to discharging stroke patients from their care. Both groups are advised to perform the given interventions for 3 times per week for 12 weeks under the supervision of their caregiver. Outcomes of interventions will be measured using timed up and go test (for mobility), ten-meter walk test (for gait speed), stroke self-efficacy questionnaire (for self-efficacy) and hospital anxiety and depression scale (for anxiety level). All data will be analyzed using descriptive and inferential statistics.

**Discussion:**

This study will provide the information on the effectiveness of HBT in comparison to UP among stroke population who are discharged from rehabilitation. Findings from the study will enable rehabilitation professionals to design effective discharge care plan for stroke survivors in combating functional decline when no longer receiving hospital-based therapy.

**Trial registration:**

Australian New Zealand Clinical Trials Registry, ACTRN12619001182189 (last updated 22/11/2019).

## 1 Introduction

Stroke is a notable global health problem and cause of disability worldwide.^[1]^ Generally, mortality rate of acute stroke has reduced with the advancement of medical care; most countries now have an increasing number of stroke survivors hence those living with disability.^[2]^ This increased prevalence of stroke-related disability which requires continuous and long-term rehabilitation and healthcare support places great burden on healthcare system.^[2]^

Rehabilitation remains the mainstay of treatment to combat post-stroke disability.^[3]^ The service is provided during hospitalization for acute and sub-acute stroke, and normally continued in out-patient settings once the stroke patient is discharged home. Physiotherapy is part of the multidisciplinary rehabilitation program that aim to improve strength, balance, coordination, endurance and flexibility of stroke patients.^[3]^ Therapy sessions for stroke are normally provided 2 to 3 times per week and continued for as long as 1 year or more post-onset depending on the patients needs.^[3]^ Shortage of manpower to cater for the fast-growing number of new stroke patients which outnumbered the discharged patients has however hinder the provision of further therapy and monitoring once the patient is discharged from hospital-based rehabilitation.^[4–6]^ In usual practice, stroke patient will be provided with home exercise program in a form of written or verbal prescription and advised to try maintain functional performance upon discharged from rehabilitation.^[4]^

Studies have shown that functional decline occurred in stroke survivors post-discharge from rehabilitation due to poor exercise adherence, lack of motivation, musculoskeletal issues and fatigue.^[7]^ Past studies that assessed the function of stroke survivors at 1 year or more post-discharge reported deterioration of gait speed and balance ability among the survivors at several months of discharge.^[8,9]^ Therefore, the continuity of rehabilitation at post-discharge episode is crucial to prevent functional deterioration in stroke survivors. There is a need to review the existing “usual practice” and develop a better approach. A few studies have shown that functional deterioration can be impeded with a rehabilitation or care intervention based at home.^[9]^ Home-based therapy (HBT) was able to cease the deterioration and improve the performance of activities of daily living (ADL) in stroke patients.^[10,11]^ Thus, HBT might be an alternative approach for stroke survivors who require long-term management. However, the overall effects of HBT on post-discharged stroke patients remain unknown due to scarcity of research on this population. Self-efficacy and emotional aspect of stroke survivors play an important role in determining quality of life in long term post-stroke,^[12]^ but no studies have evaluated the effectiveness of HBT with regards to these outcomes. Therefore, the purpose of this study is to determine the effectiveness of HBT in stroke survivors following discharge from rehabilitation in term of functional outcome (mobility and gait speed), self-efficacy and anxiety level. This study also aims to assess the feasibility of HBT plan on this population.

## 2 Methods

### 2.1 Study design and setting

**Figure 1:**
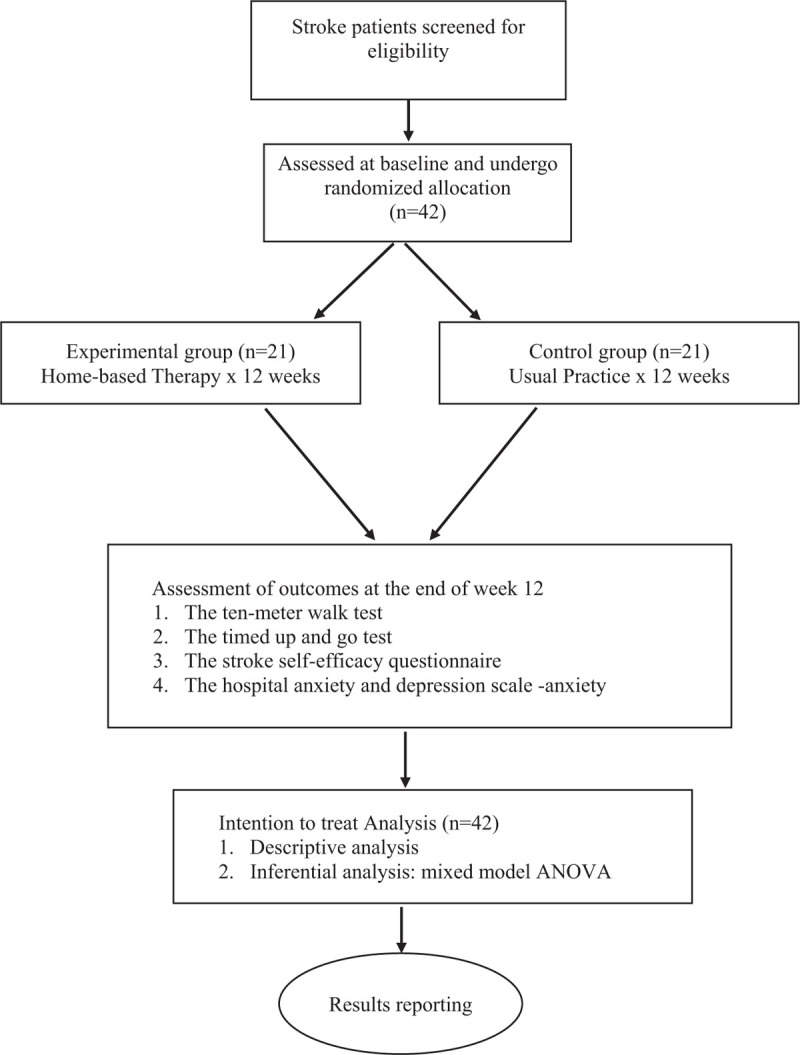
Flow of the Study.

This is an assessor-blinded randomized control trial comparing 2 types of intervention which are home-based therapy (experimental group) and usual practice (control group). The study will be conducted at 2 public rehabilitation hospitals, namely Universiti Kebangsaan Malaysia Medical Center and Cheras Rehabilitation Hospital. Both hospitals are main referral centers for stroke cases in Kuala Lumpur, the capital of Malaysia. List of all stroke patients to be discharged from the 2 hospitals following rehabilitation will be used to recruit participants adequately. Figure 1 shows the flow of the study.

### 2.2 Study participants

Potential participants will be recruited by the main researcher; inclusion criteria are as follows:

1. Clinically diagnosed as stroke by a medical officer.
2. Completed hospital-based rehabilitation and scheduled for discharge.
3. Age 18 to 80 years old. (to minimise risk of frailty which is prevalent in those above 80)^[13]^
4. Availability of carer to monitor home-based interventions.
5. Score 3 or more on functional ambulation categories (FAC) (3: ambulator-dependent for supervision; 4: ambulator-independent level surface only; 5: ambulator-independent)

Exclusion criteria are:

1. Score below 20 on Montreal Cognitive Assessment (MoCA) which indicates presence of cognitive impairment.
2. Score 4 or more on a Modified Rankin Scale (4: moderate severe disability; 5: severe disability; 6: death).
3. Attended by a home physiotherapist after discharged from HOS.
4. Score above 11 on Hospital anxiety and depression scale -depression subscale (HADS-D) which indicates moderate to severe depression.
5. Presence of other medical conditions such as severe neurological and musculoskeletal disorders or comorbidities.

### 2.3 Participants allocation

Stroke survivors will be randomized into either HBT (experimental group) or UP (control group) using stratified randomization method, with the use of microsoft-excel 2016 software by an independent researcher. Stratification variables are age, consisting of adult (18–59 years), young-old (60–74 years) and old-old (75–85 years), and disability level, namely 1 (no significant disability), 2 (slight disability) and 3 (moderate disability). Using the software, a random number column of subjects will be generated, and by using the sort function, recruited subjects will be stratified according to the sequence of age, disability level and random number. Subjects are then alternately allocated into each trial arm according to their age and disability level categories. Therefore, both the HBT and UP trial arm will consist of subjects with the different age and disability levels.

### 2.4 Interventions

The interventions in this study will be conducted for 12 weeks. HBT group will receive home-based exercise program consisting of 9 strengthening, walking, task-related and balance exercises, which aim to improve muscle strength, balance and gait ability. The exercises were designed based on a protocol reported in a previous study^[14]^ and using the frequency, intensity, time and type (FITT) principle.^[15]^ The total duration of the exercise program is between 45 to 60 minutes, depending on the participants ability. Participants will be instructed to perform the given exercises at a comfortable pace for 3 times per week, monitored or assisted by their caregivers. Training session will be provided to all participants and caregivers by the main researcher prior to implementing home-based sessions. Logbook will be given to all participants to document their home-based exercise sessions. The graphic and instructions for exercises in the logbook were adopted from an exercise website (https://www.physiotherapyexercises.com/). Once per 2 weeks telephone call will be attempted for each participant to monitor their exercise adherence and occurrence of any potential adverse effects related to exercise such as muscle soreness and cramps. Exercises will be progressed with regards to intensity for participants who report no adverse effects. Table 1 shows the home-based exercise program to be provided to participants in HBT group.

**Table 1:** Home-based exercise program for HBT group.

Warm up exercise (stretching exercise)
• Head turn with over pressure
• Neck lateral flexor stretch
• Posterior shoulder stretch
• Trunk rotation
• Hamstring stretch in sitting
• Seated gluteal stretch
1. Sit to stand exercise – any one exercise from the list below according to participant's standing balance and lower limb muscle strength:
• Sit to stand with support
- participant with poor standing balance
• Sit to stand without support
- participant with fair/good standing balance and muscle strength 3/5
• Sit to stand in tandem position
- participant with good standing balance and muscle strength above 4/5
• Sit to stand on a low stool
- advance participant with good standing balance and muscle strength above 4/5
2. Stepping in different directions
3. Stepping up and down stairs or a block
4. Reach to target exercise – any one exercise from the list below according to participants standing balance:
• Stand and reach to targets
- participant with fair standing balance
• Step and reach to targets
- participant with good standing balance
• Sit and reach to targets
- participant with poor standing balance
5. Walk and pivot turn exercise
6. Sideway walking exercise – either one exercise from below according to participants standing balance
• Cross over side step
- participant with good standing balance
• Walk sideway
- participant with fair/good standing balance
7. Walk with stepping exercise – either one exercise from below according to participants standing balance and lower limb muscle strength
• Walking Lunge
- participant with fair/good standing balance and muscle strength above 3/5
• Walking with big step
- participant with fair standing balance and muscle strength above 3/5
8. Wall push-ups
9. Arm Exercise – any one exercise from the list below according to participants upper limb muscle tone and strength
• Upper limb proprioception exercise
- participant with muscle tone above 1+
• Upper limb active range of exercise
- participant with muscle tone above 1 and muscle strength below 3/5
• Upper limb strengthening exercise
- participant with normal muscle tone and muscle strength 3 and above

HBT = home-based therapy.

Meanwhile participants in UP group will be advised to perform exercises as prescribed by the therapist prior to discharged from physiotherapy service. This is the common practice among the attending therapists when discharging stroke patients. Similarly, participants will be monitored in term of exercise adherence and occurrence of any potential adverse effects related to exercise such as muscle soreness and cramps via telephone call once in every 2 weeks. Participants will also be asked to record their exercise practice in a logbook.

### 2.5 Outcomes

Feasibility of both interventions will be assessed with regards to patients acceptance and adherence, and the occurrence of adverse effect.

1. Stroke survivors acceptance will be collected during post-trial assessment with question of “What do you think about this home-based therapy?”. Response from the survivors will be categorized into positive and negative responses and analysed descriptively.
2. Adherence rate will be reported in term of percentages of practice (number of sessions performed out of the total sessions required i.e., 36 sessions). Number of practice sessions performed will be obtained from participants logbook.
3. Occurrence of adverse effects related to exercise such as muscle soreness, cramps and muscle strain will be monitored through telephone call (once every 2 weeks) and during post-trial assessment.

The intended outcomes of the interventions in this study are:

1. Functional performance
  - Time up and go (TUG) test will be used to assess mobility level and falls risk. The cutoff point of high risk of fall for stroke survivor is >14 seconds.^[16]^ TUG was shown to have high test-retest reliability (ICC = 0.96) and excellent correlation with the comfortable gait speed test (r = −0.86), fast gait speed test (r = −0.91), ascending stair climbing test (r = 0.86), descending stair climbing test (r = 0.90) and 6-minute walk test (r = 0.92).^[17]^ Participants will be instructed to stand up from a standard chair and walk safely as quickly as possible for 3-meter, turn around and walk back to the chair and sit down. The timing begins at the instruction “Go” and stops when participants sit down on the chair.
  - The 10-meter walk test (10mWT) will be used to assess walking speed among participants and determine the functional ambulatory classification of each participants. The participant will be instructed to walk along the 10-meter walkway and stop at the end point with comfortable speed. The middle of the 6-meter walkway will be timed as the participant walks. The test will be repeated with fast walking speed. The 10mWT has high test-retest reliability (ICC = 0.95–0.99) in stroke survivors.^[18]^
2. Self-efficacy: The stroke self-efficacy questionnaire (SSEQ), a 13-item self-administered questionnaire which designed specifically for stroke survivors will be used to assess the level of self-efficacy among the study participants. SSEQ consists of two self-efficacy domains: activity (items 1 to 8) and self-management (items 9 to 13).^[19]^ Participants need to rate their confidence level on a 3-point scale, from 0 which indicates “not at all confident” to 3 which represents “very confident”. The total score of SSEQ will be calculated by summation of each item score. Higher score of SSEQ indicates higher self-efficacy level.^[19]^ SSEQ has good reliability, with Cronbach α coefficient value 0.90 and person separation index (PSI) > 0.80 for both activity and self-management domains.^[19]^
3. Anxiety level: Hospital anxiety and depression scale (HADS)^[20]^ will be used to measure anxiety level of the patient. HADS is divided into an anxiety subscale (HADS-A) and a depression subscale (HADS-D), each subscale contains 7 questions and can be calculated separately. HADS-A will be used to assess the anxiety level among participants at baseline and post-trial. The cut-off score for anxiety using HADS-A is 8 and above, with specificity of 0.78 and a sensitivity of 0.9. Bjelland et al^[21]^ who reviewed HADS in 747 studies reported that HADS-A has good reliability value, indicated by Cronbach's α coefficient between 0.68 to 0.93.

### 2.6 Assessment of outcomes

All participants will be assessed at baseline and at the end of week 12 of interventions by a therapist who is blinded to the group allocation and trained to conduct the standardized tests. To avoid recall bias, the recorded baseline assessment data will not be accessible to the blinded assessor during the post-trial assessment.

### 2.7 Sample size

GPower software version 3.0.10 was used to estimate required sample size for this study. This study used mixed model analysis of variance (ANOVA) to analyze time, group and interaction effects of the interventions. Therefore F-test (ANOVA repeated measure, within-between interactions) were chosen. The study power was set at 80% and alpha value set at 0.05. Based on these, a minimum sample of 42 subjects is required, i.e., 21 participants in each arm.

### 2.8 Data analysis

All data will be entered into IBM Statistical Packages for Social Sciences (SPSS) version 23.0. Intention-to-treat (ITT) method will be used; all the participants recruited at baseline will be included in the outcome analysis. Using ITT, missing data will be replaced with last observation carried forward. Socio-demography and health profile of the participants will be analyzed descriptively and reported as frequencies (percentages) and mean (standard deviation) or median (inter-quartile range). The effects of the interventions will be analyzed using mixed model ANOVA and reported as main time, group and time-group interaction effects for each intervention outcome. Level of significance will be set as *P* < .05 for all results.

### 2.9 Ethics and dissemination

This study received ethical approval from the Research and Ethics Committee of Universiti Kebangsaan Malaysia and the National Medical Ethics and Research Committee (NMERC) (study ID: NMR–19–941–46993). NMERC, being an independent research committee under the Malaysia Ministry of Health is also responsible in monitoring the study progress. All participants will be asked to provide an informed consent prior to participating in the study by the main researcher. Participants can withdraw anytime during the study without providing an explanation. Participants personal information and data will be kept confidential. Investigators in this study will have access to the final trial dataset. The findings of this study will only be published in a peer-reviewed journal.

## 3 Discussions

The decline in stroke survivors functional performance following discharge from formal rehabilitation place the survivors at increased risk of stroke recurrence, falls and emotional disturbances which eventually lead to reduced quality of life.^[22,23]^ As such, there is a need to ensure the continuity of therapy to prevent these negative consequences among the stroke survivors. Due to the high workload and manpower shortage in many rehabilitation centers particularly in developing countries, extended stroke rehabilitation in hospital settings is difficult. Many stroke survivors are discharged rather prematurely when further functional improvement is still possible with regular rehabilitation therapies. There is also no monitoring of functional performance once the stroke survivors are discharged from rehabilitation service, raising concern on the needs for proper discharge care plan.

Training stroke survivors and caregivers to perform structured home therapy which focuses on therapeutic exercises may be a beneficial strategy to fill these needs. Studies have shown that home-based therapy can be as effective as hospital-based program in preventing functional deterioration of stroke survivors^[11,24,25]^ However, evidence on its effectiveness, particularly among post-discharged stroke patients are still limited to inform clinical practice, which warrants more studies on this topic area. Further, past studies have not included psycho-behavioral and emotional aspects, 2 important determinants of quality of life among stroke survivors^[26]^ in their assessment of intervention outcome. Therefore, this study is proposed to fill these gaps in knowledge and strengthen evidence on the benefit of home-based therapy for stroke survivors. This study will also compare home-based therapy with the existing common practice for discharged stroke survivors and provide information whether HBT is a better approach for this group of population in preventing long-term functional problems. It is our hope that findings from this study can be used to enable rehabilitation professionals to design an effective care plan for stroke patients who are scheduled for discharge and as a reference for future studies regarding home-based therapy and post-discharge care.

## Acknowledgments

The authors thank the Research and Ethics Committee of Universiti Kebangsaan Malaysia (UKMMC) and National Medical Research and Ethics Committee, Ministry of Health for the study approval.

## Author contributions

**Conceptualization:** Chong Pui Kei and Nor Azlin Mohd Nordin.

**Funding acquisition:** Nor Azlin Mohd Nordin.

**Methodology:** Chong Pui Kei and Nor Azlin Mohd Nordin.

**Supervision:** Nor Azlin Mohd Nordin, Aznida Firzah Abdul Aziz.

**Writing – original draft:** Chong Pui Kei.

**Writing – review & editing:** Nor Azlin Mohd Nordin, Aznida Firzah Abdul Aziz.
